# Predictors for diabetes and hypertension among bus drivers and conductors in South India

**DOI:** 10.6026/973206300200495

**Published:** 2024-05-31

**Authors:** Kamal Batcha Mohamed Ali, Selvaraju Sathish Kumar, Perumal Kandasami Govindarajan, Kamal Batcha Mujibur Rahman, Sebastian Nancy

**Affiliations:** 1 Department of Community Medicine, Vinayaka Mission's Medical College and Hospital, Vinayaka Mission's Research Foundation - Deemed to be University (VMRF-DU), Karaikal, Puducherry, India

**Keywords:** drivers, conductors, transport workers, diabetes, hypertension, non-communicable diseases

## Abstract

Bus drivers and conductors are facing various health hazards due to stressful working conditions. They are exposed to various occupational hazards which lead to deterioration of their health over a period of time. Therefore, it is of interest to evaluate the prevalence of diabetes and hypertension among bus drivers and conductors and to determine the factors associated with diabetes and hypertension. This cross-sectional study was done among 293 bus drivers and 157 conductors during March 2018 to December 2018 and the data was collected using a semi structured questionnaire after obtaining informed consent. Each individual was investigated for Blood sugar and Blood Pressure. Out of 450 study participants, about 6.9% were diabetic and 50.2% were hypertensive. Transport workers with single marital status, those who belong to rural areas and drivers were significant predictors for diabetes. Overweight was significantly associated with the Diabetes in negative direction. Marital status, years of experience and anxiety were significantly associated with hypertension. Hemoglobin level, total cholesterol level and blood urea level also emerged as predictors for Hypertension. Non-communicable diseases like diabetes and hypertension have surpassed the communicable diseases in affecting the health of people with distinct occupations like bus drivers and conductors.

## Background:

Bus drivers and conductors are facing various health hazards due to their stressful working conditions [1]. They are exposed to various occupational hazards which lead to deterioration of their health over a period of time [1]. Bus driving is a classic example of high-strain occupation, with high risks of physical and mental occupational defense-lessness, leading to absenteeism and decreased productivity of employees and enterprises [1]. Prevalence of morbidities is more in bus drivers and conductors than general population and the health hazards are predominantly non-communicable diseases [2]. Road transport drivers are one of the professional groups whose activities have a strong impact of public safety [2]. In view of the natural professional activity, the drivers are at a higher risk of obesity, hypertension and hyperlipidemia, and carbohydrate metabolism disorders such as diabetes mellitus [2,3]. Diabetes mellitus (DM) refers to a group of common metabolic disorders in which a person has high blood sugar, either because the pancreas does not produce enough insulin, or because cells do not respond to the insulin that is produced [4]. Several distinct types of DM are caused by a complex interaction of genetics and environmental factors [5]. Both complications of diabetes and medications' side effects can affect driving skills [5]. Diabetic neuropathy and retinopathy are two common complications which can cause muscle weakness and amputation [6]. Moreover, treatment of diabetes can result in hypoglycemia, which in turn, may lead to increased reaction time, imbalance and loss of consciousness [6,7]. Drivers are faced with some health hazards in their job, such as stress, sitting for long periods, night and rotatory shifts and that increases risk for obesity and hypertension which are well-known risk factors for diabetes [5,6]. High prevalence of excessive body weight, high blood pressure and hyperlipidemia are risk factors for diabetes mellitus in professional drivers that indicates a need to undertake multidimensional actions on this particular profession and there is a dire need to involve various health care sectors [1, 2]. The prevalence of hypertension is high among bus drivers [7]. Age > 35 years, elevated BMI, supporting a large family, and dietary habits associated with the job showed significant association with hypertension [7]. Primary and secondary prevention strategies need to be emphasized in this occupational group [8]. All groups of professional drivers especially those carrying passengers are at excess risk of hypertension, myocardial infarction, and hemorrhagic stroke [8]. Occupational bus drivers in a developing country like India deserve special concern. They have to be extremely careful in handling heavy vehicles laden with passengers [9]. Traffic congestion, exposure to vehicle exhausts, constant whole-body vibration, poor condition of roads, poor town planning and traffic regulation, over speeding due to competition between buses, and carelessness of pedestrians contribute to their misery [3,4]. Besides, most of the drivers are in the habit of eating main meals from hotels and consuming snacks (often oily and fried) and fast-food items between trips. Many resort to alcohol and smoking to overcome stress [5]. Consequently, they have an additional risk of developing hypertension [6]. Prophylactic and detailed pre-placement examinations should be considered, depending on the rate and the intensity of disorders [10]. These should be coupled with an introduction of primary and secondary prophylactic activities and monitoring of relevant treatment [10]. Therefore, it is of interest to identify the proportion of Diabetes and Hypertension among bus drivers and conductors and to determine the factors associated with Diabetes and Hypertension.

## Methodology:

A cross-sectional study was conducted among the bus drivers and conductors working in Government Transport Department and Private Transports of Karaikal divisions in Pondicherry. In a study on Bus drivers in Mumbai city, the prevalence of Hypertension was found to be 24.28%; taking absolute error of margin as 4%, sample size was calculated as 441. So, the minimum sample size required for the study was 450. A registered list of Drivers and Conductors was the sampling frame. The sampling frame contained all the details of individuals like name, their registration number, their contact information, telephone number and other additional information related to their enrollment in the Transport division. After obtaining the registered list of Drivers and Conductors from the concerned authority, the participants were selected from the study population by simple random sampling with the help of random number tables. The individual members were the sampling unit in this study. A pre-tested standardized semi-structured schedule was developed reviewing the questionnaires which have been used in the similar earlier studies and from different articles related to Occupational Hazards and Non-Communicable Diseases like STEPS questionnaire and WHO Occupational Health Manual. The purpose of the study was explained to all the participants and they were assured of confidentiality. After obtaining informed consent for participation in the study, the schedule was applied. The schedule was used to collect information regarding socioeconomic status, demographic and behavioural characteristics. Then blood pressure measurement, following which blood samples were, collected for random blood sugar, Haemoglobin, Blood Urea, Blood Creatinine and Total Cholesterol levels.

## Blood Pressure Measurement:

Blood pressure (BP) was measured according to seventh report by Joint National Committee on Prevention, Detection, Evaluation, and Treatment of High Blood Pressure (JNC 8) guidelines. Before measuring BP, participants were asked not to consume tea or coffee and avoid smoking, any physical activity, for at least 30 minutes before examination. They were also advised to have an empty bladder. Participants were initially seated quietly for 5 minutes in a chair with feet on the floor, and arms supported at heart level. Then, BP was measured twice in a seated position using a standard mercury sphygmomanometer.

An appropriate cuff was chosen so that 80% of the arm was encircled with the arm supported at the heart level. Two measurements were made and the average was recorded. The palpated radial pulse obliteration pressure was used to estimate Systolic Blood Pressure (SBP). The cuff was then inflated 20-30 mmHg above this level for the auscultatory determinations. The cuff deflation rate for auscultatory readings was 2 mmHg per second. SBP was recorded as the point at which the first of two or more Korotkoff sounds is heard (onset of phase 1), and the disappearance of Korotkoff sound (onset of phase 5) was used to define Diastolic Blood Pressure (DBP).

## Serum Glucose Estimation:

## Method:

GOD-POD method, End Point.

## Principle:

Glucose is oxidized by glucose oxidase (GOD) to produce gluconate and hydrogen peroxide. The hydrogen peroxide is then oxidatively coupled with 4 amino- antipyrene (4-AAP) and phenol in the presence of peroxidase (POD) to yield a red quinoeimine dye that is measured at 505 nm. The absorbance at 505 nm is proportional to concentration of glucose in the sample. Absorbance of the coloured solution is directly proportional to the glucose concentration, when measured at 505 nm.

## Reagent Composition:

Reagent 1:

Glucose oxidase - 20000 u/L

Peroxidase - 1200 u/L 

4-AAP - 0.246 mmol/L 

Reagent 2:

Glucose standard - 100 mg/dL

## Procedure:

One reagent blank and one standard were sufficient for each assay series.

Pipetting done into respective test tubes as follows:

Particulars - Blank - Standard - Sample

Reagent 1 - 1000µL 1000µL - 1000µL

Reagent 2 - ------- 10µL -------

Sample - ------- ------- 10µL

The test tubes were mixed well and incubated for 15 minutes at room temperature. The absorbance of standard and sample against reagent blank at 505 nm were measured.

## Data analysis:

All the data was initially entered to Microsoft Excel 2010 and later these spreadsheets were used for analysis. Statistical analysis was done using SPSS version 20.0. Descriptive statistics were calculated as frequency, percentage, mean and standard deviation, median and inter-quartile range. Taking presence of Hypertension and Diabetes individually as a dichotomous variable, logistic regression analysis was used. Initially, a bivariate analysis was done to ascertain the relationship of dependent variable with other variables. Then, all the variables found to be significant in bivariate analysis were entered into a multivariate logistic regression analysis (LINK FUNCTION = LOGISTIC) with various models in a nested manner. P value of < 0.05 was considered to determine significant association between two variables.

## Ethical consideration:

The study was carried out after obtaining approval from the Research Committee and Institutional Ethics Committee (EC approval number: 21/2017).

## Results:

## Socio demographic characteristics between drivers and conductors:

In the study population, about 162 (36%) transport workers were in the age group of 35 to 44 years. More than half, 230 (51.1%) drivers and conductors were hailing from rural areas. Nearly 187 (41.6%) workers received higher secondary education. Almost 401 (89.1%) workers were married. Notably, 292 (64.9%) drivers and conductors were employed in government sector and 325 (72.2%) workers were working in long distance transports. Almost 186 (41.3%) transport workers had more than 15 years' experience. (Table 1)

## Bivariate and Multivariate logistic regression models of diabetes:

In Bivariate and Multivariate Logistic Regression analysis, socio demographic variables like marital status, place of living and occupation had shown statistically significant (p<0.05) association with Diabetes. The study population with single marital status, those who reside in rural areas and drivers were associated with Diabetes. Presence of overweight was significantly (p<0.05) associated with the Diabetes in negative direction. (Table 2)

## Bivariate and Multivariate logistic regression models of Hypertension:

In Bivariate Logistic Regression analysis, socio demographic variables like marital status, type of employment and years of experience had shown statistically significant (p<0.05) association with Hypertension. The study population with single marital status, those who were employed in private sector and those with 1 to 1.5 years of experience were associated with Hypertension. Hemoglobin level, Total Cholesterol level and Blood Urea level were significantly (p<0.05) associated with Hypertension. Presence of Depression and Anxiety were also significantly (p<0.05) associated with the Hypertension. In Multivariate Logistic Regression analysis, socio demographic variables like marital status and years of experience had shown statistically significant (p<0.05) association with Hypertension. The study population with single marital status, those with 1 to 1.5 years, 5.1-10 years, 10.1-15 years and >15 years of experience were associated with Hypertension. Hemoglobin level, Total Cholesterol level and Blood Urea level were significantly (p<0.05) associated with the Hypertension. Presence of Anxiety was significantly (p<0.05) associated with the Hypertension. (Table 3)

## Discussion:

Data shows that 6.9% participants were diabetic and 50.2% were hypertensive. Transport workers with single marital status, those who belong to rural areas and drivers were significant predictors for Diabetes. Overweight was significantly associated with the Diabetes in negative direction. Socio demographic variables like marital status and years of experience were significantly associated with Hypertension. Hemoglobin level, Total Cholesterol level and Blood Urea level also emerged as predictors for Hypertension. Anxiety was significantly associated with Hypertension. In current study, 6.9% of the participants were found to be Diabetic and 8.2% were pre diabetic. Road transport drivers are one of the professional groups whose activities have a strong impact of public safety. In view of the natural professional activity, the drivers are at a higher risk of obesity, hypertension and hyperlipidemia, and carbohydrate metabolism disorders such as diabetes mellitus [11]. Diabetes mellitus (DM) refers to a group of common metabolic disorders in which a person has high blood sugar, either because the pancreas does not produce enough insulin, or because cells do not respond to the insulin that is produced. Several distinct types of DM are caused by a complex interaction of genetics and environmental factors. Both complications of diabetes and medications' side effects can affect driving skills [12]. Diabetic neuropathy and retinopathy are two common complications which can cause muscle weakness or even amputation [12]. Moreover, treatment of diabetes can result in hypoglycemia, which in turn, may lead to increased reaction time, imbalance and loss of consciousness [13]. In one study carried out on Hong Kong professional drivers, the prevalence of diabetes was 8.1% [14], while in another study; this prevalence was 7% [15]. It can be suggested that drivers are faced with some health hazards in their job, such as stress, sitting for long periods, night and rotatory shifts put them at a higher risk for obesity and hypertension which are well-known risk factors for diabetes.

In a study, hyperglycemia was found in 52.1% of the drivers, 9.1% of them were in diabetic stage, and with HbA1C criteria 77.6% of these drivers were in this stage [16]. High prevalence of excessive body weight and high blood pressure and hyperlipidemia are risk factors for diabetes mellitus in professional drivers that indicates a need to undertake multidimensional actions target on this particular profession and involving various health care sectors [16]. Hypertension plays an important part in deteriorating their positive health leading to sickness absenteeism [17, 18]. The administrative authorities should take necessary steps for the welfare of health of the transport workers. Henceforth the study findings also suggest a separate unit to look after the welfare of bus conductors and bus drivers' health along with safety measures. About 50.2% of current study populations were hypertensives and 32.7% were in the pre-hypertensive stage. In the study by Taklikar *et al.*, hypertension was seen among 24%, Dyspepsia, regurgitation among 52%, lower back among 79% of bus drivers [18]. In their study, Blood pressure was significantly high among bus drivers having high stress score. High blood pressure was recorded in 16.4% of drivers in another study [18].

Prevalence of hypertension was high among bus drivers. Age >35 years, elevated BMI, supporting a large family, and dietary habits associated with the job showed significant association with hypertension. Primary and secondary prevention strategies need to be emphasized in this occupational group. Among 179 bus drivers studied, 16.8% (30/179) had normal BP, 41.9% (75/179) had prehypertension, and 41.3% (74/179) had hypertension. Isolated systolic HTN was seen in 6.70% (12/179) individuals [19]. Out of 74 hypertensive, 9 (12.1%) were aware of their hypertension, while 3 (4.0%) were medicated and only 1 (1.3%) had BP adequately controlled in the study by Lakshman *et al.* [19]. Systolic BP and Diastolic BP were significantly higher among the bus drivers when compared to the controls [20]. There was a significant positive correlation between exposure level and systolic and diastolic blood pressure [20]. Many resort to alcohol and smoking to overcome stress. It follows logically that they may have an additional risk of developing HTN [21]. In addition, prolonged exposure to high intensity of sound can increase the blood pressure among the bus drivers [21].

## Conclusion:

Drivers and conductors showed increased risk factor profiles for non-communicable diseases like diabetes and hypertension and hence they are considered as a vulnerable group and require specific attention pertaining to their health care problems. Promotion of specific preventive strategies including risk factor surveillance is the need of the hour. Prophylactic and detailed pre-placement examinations should be considered, depending on the rate and the intensity of disorders. These should be coupled with an introduction of primary and secondary prophylactic activities and monitoring of relevant treatment.

## Financial support and sponsorship:

NIL

## Figures and Tables

**Figure 1:**
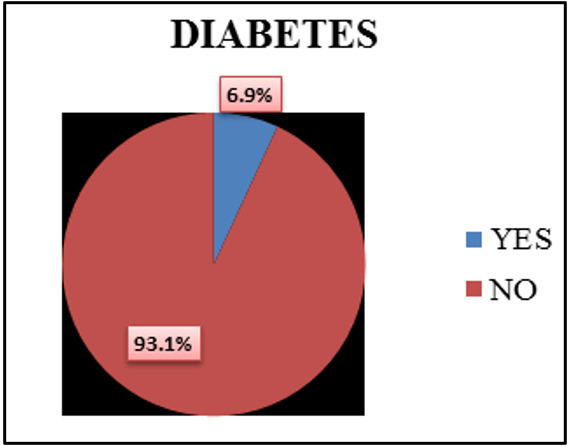
Pie diagram showing Diabetes distribution among transport workers (N = 450)

**Figure 2:**
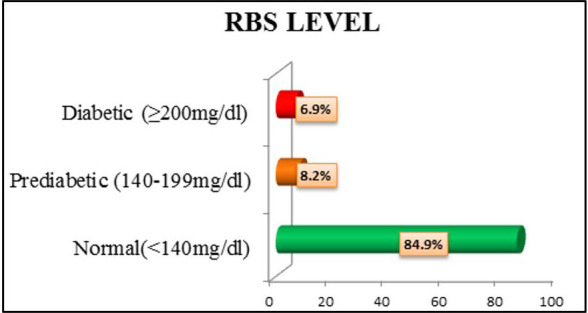
Bar diagram showing Random Blood Sugar levels among transport workers (N = 450)

**Figure 3:**
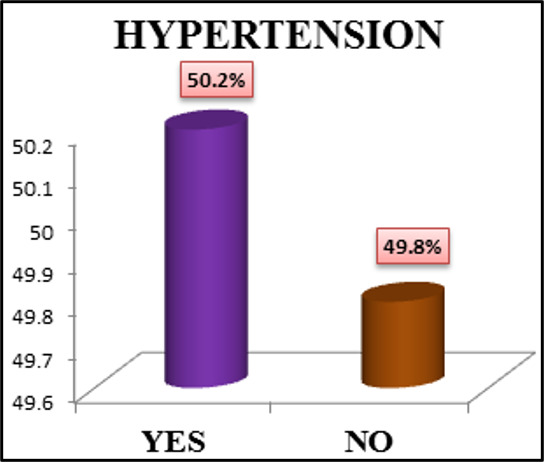
Bar diagram showing Hypertension distribution among transport workers (N = 450)

**Figure 4:**
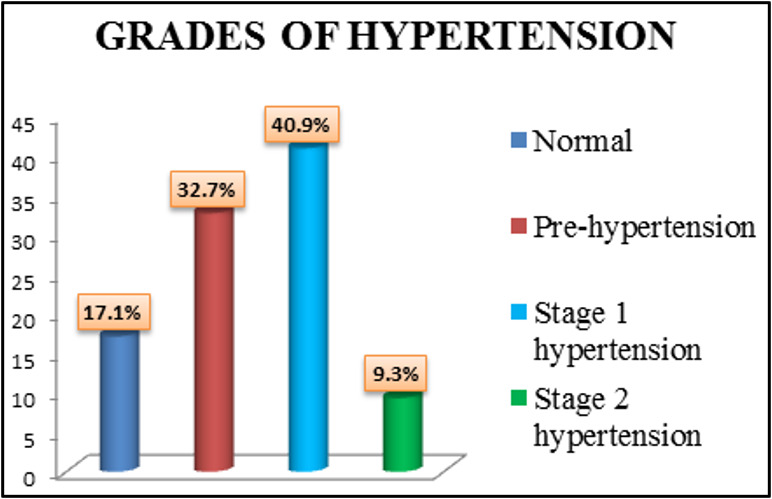
Bar diagram showing Grades of Hypertension among transport workers (N = 450)

**Table 1:** Description of socio demographic characteristics between drivers and conductors (N=450)

**S.No**	**Variables**	**Drivers**	**Conductors**	**total**
**1.**	**Age group**			
	<25	2 (0.7%)	12 (7.6%)	14 (3.1%)
	25-34	55 (18.8%)	26 (16.6%)	81 (18.0%)
	35-44	111 (37.9%)	51 (32.5%)	162 (36.0%)
	45-54	88 (30.0%)	61 (38.9%)	149 (33.1%)
	>54	37 (12.6%)	7 (4.5%)	44 (9.8%)
**2.**	**Residence**			
	Urban	156 (53.2%)	64 (40.8%)	220 (48.9%)
	Rural	137 (46.8%)	93 (59.2%)	230 (51.1%)
**3.**	**Education**			
	Primary	2 (0.7%)	4 (2.5%)	6 (1.3%)
	Secondary	79 (27.0%)	49 (31.2%)	128 (28.4%)
	Higher secondary	138 (47.1%)	49 (31.2%)	187 (41.6%)
	Graduate	74 (25.3%)	55 (35.0%)	129(28.7%)
**4.**	**Marital status**			
	Married	260 (88.7%)	141 (89.8%)	401 (89.1%)
	Single	24 (8.2%)	16 (10.2%)	40 (8.9%)
	Divorced	5 (1.7%)	0 (0%)	5 (1.1%)
	Separated	4 (1.4%)	0 (0%)	4 (0.9%)
**5.**	**Employment**			
	Government	189 (64.5%)	103 (65.6%)	292 (64.9%)
	Private	104 (35.5%)	54 (34.4%)	158 (35.1%)
**6.**	**Bus route type**			
	Mofussil	45 (15.4%)	70 (44.6%)	115 (25.6%)
	Long distance	247 (84.3%)	78 (49.7%)	325 (72.2%)
	School bus	1 (0.3%)	9 (5.7%)	10 (2.2%)
**7.**	**Experience**			
	≥1 year	65 (22.2%)	51 (32.5%)	116 (25.8%)
	1.1-5 years	39 (13.3%)	13 (8.3%)	52 (11.6%)
	5.1-10 years	23 (7.8%)	17 (10.8%)	40 (8.9%)
	10.1-15 years	38 (13.0%)	18 (11.5%)	56 (12.4%)
	>15 years	128 (43.7%)	58 (36.9%)	186 (41.3%)

**Table 2:** Bivariate and Multivariate logistic regression models of Diabetes among the study population (N=450)

**Independent Variable**	**Diabetes**	**OR**	**P value**	**AOR**	**P value**
	**n (%)**	**(95% CI)**		**(95% CI)**	
**AGE**					
(continuous variable)	----------	1.04	0.076		
		(0.10-1.09)			
PLACE OF LIVING					
Rural	23 (10.0)	2.94	0.010*	3.21	0.009*
-230		(1.28-6.73)		(1.33 - 7.77)	
Urban (Ref)	8 (3.6)	1		1	
-220					
EDUCATION					
Primary	6 (100)	2.61	0.998		
-6		(0.56-3.25)			
Secondary	8 (6.3)	1.78	0.996		
-128		(0.23-2.01)			
Higher secondary	17 (9.1)	1.62	0.996		
-187		(0.20-1.92)			
Graduate (Ref) (129)	0 (0)	1			
MARITAL STATUS					
Single	6 (15.0)	2.65	0.046*	2.93	0.047*
-40		(1.01-6.91)		(1.01 - 8.51)	
Divorced	0 (0)	0	0.999	0	0.999
-5		(0.00-0.00)		(0.00-000)	
Separated	0 (0)	0	0.999	0	0.999
-4		(0.00-0.00)		(0.00-000)	
Married (Ref)	25 (6.2)	1		1	
-401					
OCCUPATION					
Driver	27 (9.2)	3.88	0.013*	5.87	0.002*
-293		(1.33-11.30)		(1.91 - 18.01)	
Conductor (Ref)	4 (2.5)	1		1	
-157					
TYPE OF EMPLOYMENT					
Private	12 (7.6)	1.18	0.664		
-158		(0.55-2.50)			
Government (Ref)	19 (6.5)	1			
-292					
EXPERIENCE					
1.1-5 years	6 (11.5)	3.65	0.053		
-52		(0.98-13.54)			
5.1-10 years	0 (0)	0	0.998		
-40		(0.00-0.00)			
10.1-15 years	6 (10.7)	3.36	0.069		
-56		(0.90-12.43)			
>15 years	15 (8.1)	2.45	0.119		
-186		(0.79-7.59)			
≤1 year (Ref)	4 (3.4)	1			
-116					
ALCOHOL CONSUMPTION					
Yes	21 (6.7)	0.9	0.798		
-314		(0.41-1.97)			
No	10 (7.4)	1			
-136					
SMOKING					
Yes	15 (7.9)	1.32	0.456		
-189		(0.63-2.74)			
No	16 (6.1)	1			
-261					
OVERWEIGHT					
Yes	21 (5.5)	0.31	0.005*	0.29	0.007*
-385		(0.14-0.70)		(0.12 - 0.71)	
No	10 (15.4)	1		1	
-65					
HYPERTENSION					
Yes	18 (8.0)	1.4	0.367		
-226		(0.67- 2.94)			
No	13 (5.8)	1			
-224					
ANXIETY					
Yes	6 (8.3)	1.28	0.598		
-72		(0.50-3.25)			
No	25 (6.6)	1			
-378					

**Table 3:** Bivariate and Multivariate logistic regression models of Hypertension among the study population (N = 450)

**Independent Variable**	**HTN n (%)**	**OR (95% CI)**	**P value**	**AOR (95% CI)**	**P value**
**AGE**					
(continuous variable)	----------	0.1 (0.98-1.02)	0.955		
PLACE OF LIVING					
Urban -220	108 (49.1)	0.91 (0.63-1.32)	0.639		
Rural (Ref) -230	118 (51.3)	1			
EDUCATION					
Primary -6	6 (100)	0.99 (0.24-5.50)	0.357		
Secondary -128	72 (56.3)	1.43 (0.87-2.34)	0.151		
Higher secondary (187)	87 (46.5)	0.97 (0.61-1.52)	0.894		
Graduate (Ref) (129)	61(47.3)	1			
MARITAL STATUS					
Single -40	29 (72.5)	2.73 (1.37-5.61)	0.006*	3.62 (1.29-10.15)	0.014*
Divorced -5	0 (0)	0 (0.00-0.00)	0.999	0 (0.00-0.00)	0.999
Separated -4	0 (0)	0 (0.00-0.00)	0.999	0 (0.00-0.00)	0.999
Married (Ref) (401)	197 (49.1)	1		1	
OCCUPATION					
Driver -293	149 (50.9)	0.9 (0.63-1.37)	0.715		
Conductor (Ref) (157)	77 (49.0)	1			
TYPE OF EMPLOYMENT					
Private -158	109 (69.0)	3.32 (2.20-5.01)	0.001*	1.32 (0.58-3.00)	0.499
Government (Ref) (292)	117 (40.1)	1		1	
BUS ROUTE TYPE					
Mofussil -115	58 (50.4)	0.25 (0.05-1.25)	0.092		
Long distance (325)	160 (49.2)	0.24 (0.05-1.15)	0.076		
School bus (Ref) (10)	8 (80.0)	1			
EXPERIENCE					
1.1-5 years -52	34 (65.4)	2.24 (1.14-4.42)	0.019*	13.27 (5.04-34.96)	0.001*
5.1-10 years -40	19 (47.5)	1.07(0.52-2.21)	0.843	4.45 (1.67-11.86)	0.003*
10.1-15 years -56	26 (46.4)	1.03 (0.54-1.95)	0.927	6.23 (2.43-15.99)	0.001*
>15 years -186	94 (50.5)	1.21 (0.76-1.93)	0.413	13.54 (5.72-32.01)	0.001*
≤1 year (Ref) (116)	53 (45.7)	1		1	
ALCOHOL CONSUMPTION					
Yes -314	165 (52.5)	1.36 (0.90-2.03)	0.134		
No -136	61 (44.9)	1			
SMOKING					
Yes -189	88 (46.6)	0.77 (0.53-1.13)	0.187		
No -261	138 (52.9)	1			
HB LEVEL					
(continuous variable)	----------	1.12 (1.03-1.22)	0.007*	0.86 (0.75-0.97)	0.020*
TOTAL CHOLESTEROL LEVEL					
(continuous variable)	----------	1.01 (1.01-1.02)	0.001*	1.02 (1.01-1.03)	0.001*
BLOOD UREA LEVEL					
(continuous variable)	----------	1.05 (1.02-1.08)	0.001*	1.03 (1.00-1.06)	0.032*
BLOOD CREATININE LEVEL					
(continuous variable)	----------	2.11 (0.98-4.50)	0.054		
DIABETES					
Yes -31	18 (58.1)	1.4 (0.67- 2.94)	0.367		
No -419	208 (49.6)	1			
OVERWEIGHT					
Yes -385	195 (50.6)	1.12 (0.66- 1.90)	0.659		
No -65	31 (47.7)	1			
DEPRESSION					
Yes -59	40 (67.8)	2.32 (1.29- 4.14)	0.005*	2.72 (1.10-6.73)	0.030*
No -391	186 (47.6)	1		1	
ANXIETY					
Yes -72	56 (77.8)	4.28 (2.37-7.73)	0.000*	1.63 (0.70-3.78)	0.256
No -378	170 (45.0)	1		1	
